# The Activity of the Neutral Sphingomyelinase Is Important in T Cell Recruitment and Directional Migration

**DOI:** 10.3389/fimmu.2017.01007

**Published:** 2017-08-21

**Authors:** Lena Collenburg, Niklas Beyersdorf, Teresa Wiese, Christoph Arenz, Essa M. Saied, Katrin Anne Becker-Flegler, Sibylle Schneider-Schaulies, Elita Avota

**Affiliations:** ^1^Institute for Virology and Immunobiology, University of Würzburg, Wuerzburg, Germany; ^2^Institute for Organic and Bioorganic Chemistry, Humboldt University of Berlin, Berlin, Germany; ^3^Chemistry Department, Faculty of Science, Suez Canal University, Ismailia, Egypt; ^4^Department of Molecular Biology, University Duisburg-Essen, Essen, Germany

**Keywords:** neutral sphingomyelinase, T cell migration, ceramide, polarization, adhesion, LFA-1

## Abstract

Breakdown of sphingomyelin as catalyzed by the activity of sphingomyelinases profoundly affects biophysical properties of cellular membranes which is particularly important with regard to compartmentalization of surface receptors and their signaling relay. As it is activated both upon TCR ligation and co-stimulation in a spatiotemporally controlled manner, the neutral sphingomyelinase (NSM) has proven to be important in T cell activation, where it appears to play a particularly important role in cytoskeletal reorganization and cell polarization. Because these are important parameters in directional T cell migration and motility in tissues, we analyzed the role of the NSM in these processes. Pharmacological inhibition of NSM interfered with early lymph node homing of T cells *in vivo* indicating that the enzyme impacts on endothelial adhesion, transendothelial migration, sensing of chemokine gradients or, at a cellular level, acquisition of a polarized phenotype. NSM inhibition reduced adhesion of T cells to TNF-α/IFN-γ activated, but not resting endothelial cells, most likely *via* inhibiting high-affinity LFA-1 clustering. NSM activity proved to be highly important in directional T cell motility in response to SDF1-α, indicating that their ability to sense and translate chemokine gradients might be NSM dependent. In fact, pharmacological or genetic NSM ablation interfered with T cell polarization both at an overall morphological level and redistribution of CXCR4 and pERM proteins on endothelial cells or fibronectin, as well as with F-actin polymerization in response to SDF1-α stimulation, indicating that efficient directional perception and signaling relay depend on NSM activity. Altogether, these data support a central role of the NSM in T cell recruitment and migration both under homeostatic and inflamed conditions by regulating polarized redistribution of receptors and their coupling to the cytoskeleton.

## Introduction

Polarization of both membrane receptors and intracellular signaling machineries is a prerequisite for directional migration of T lymphocytes, and this is induced by relaying of signals provided by chemoattractants or membrane bound receptors. These are particularly important upon interaction with inflamed endothelia that provide docking, adherence, and polarizing signals to enable integrin activation and subsequent transmigration (1–5).

These processes are highly dependent on regulated sorting of receptors to cellular subdomains, such as the leading edge (LE), the midbody or the uropod, and dynamic reorganization of the actin cytoskeleton which may be coupled to surface receptors such as CXCR4 or LFA-1 by linker proteins, including ezrin/radixin/moesin (ERM) family proteins or talin (6–8). Furthermore, T cell polarization is characterized by compartmentalization of small GTPases such as Cdc42 to the LE to, e.g., enhance integrin affinity or induce actin polymerization for forward movement, which is further supported by uropod contraction promoted by RhoA activity in the uropod (9–11). In addition to coordinated protein sorting, T cell polarization and motility rely on membrane dynamics, for instance, by membrane supply to the LE and formation of actin-based protrusions there. Similarly, dynamic alterations of local membrane domains are important for the organization and maintenance of membrane domains essentially sorting receptors needed for adhesion, signal perception, and cytoskeletal coupling (12, 13).

Regulation of cytoskeletal dynamics has been reported in the context of ceramide accumulation after activation of sphingomyelinases (SMases) (14, 15). These catalyze sphingomyelin breakdown in response to various stimuli, also including ligation of death or inflammatory cytokine receptors (16–18). Ceramides released by the activity of the acid sphingomyelinase (ASM) condense into ceramide-enriched membrane domains that, if at the plasma membrane, are sites of endocytosis and vesicle shedding, and are also important in lysosomal maturation (19, 20) and function as well as cytoplasmic vesicle fusion (21–23). The impact of ASM activation on actin cytoskeletal reorganization is dependent on the mode of induction, compartment, and cell type with both stimulation of actin polymerization and collapse of actin-based protrusions having been reported (14, 24, 25). A more recent study established a role of the ASM for T cell transmigration in brain endothelial cells and did not, however, address the potential contribution of the enzyme in T cells (26).

In T cells, activation of the neutral sphingomyelinase (NSM2) after ligation of CD3 alone or in combination with CD28 has been described (27, 28). Ablation of NSM2 appeared to substantially enhance T cell responses such as proliferation, and, at the cytoskeletal level, adhesion to and spreading on co-stimulatory slides, while spatiotemporal overactivation of the enzyme by measles virus causes cytoskeletal paralysis. These observations clearly support a major role of this sphingomyelin metabolizing enzyme in the regulation of stimulated reorganization of the actin cytoskeleton under physiologic conditions (28).

Indicating that the NSM2 may play a role in directional migration of leukocytes, this was lost upon pharmacologic NSM2 inhibition (29). The impact of the enzyme on T cell migration and motility has so far not been addressed. Using both pharmacologic and genetic inhibitory approaches, we established that the NSM indeed contributes to directional T cell migration *in vivo* and *in vitro*, where the enzyme proved to be particularly important in restrictive environments. In the absence of NSM activity, T cells were largely unable to polarize their cytoskeleton and cytoskeletal organizers in response to chemokine receptor signaling, β1 integrin ligation, or contact with activated endothelial cells, and this was associated with impaired LFA-1 activation, clustering, and endothelial adhesion. By contrast, T cell transmigration appeared to require ASM rather than NSM activity indicating that both enzymes play important, yet complementary roles in this process.

## Results

### NSM Regulates the Efficiency of CD4^+^ T Cell Homing into Secondary Lymphoid Tissues

**Figure 1:**
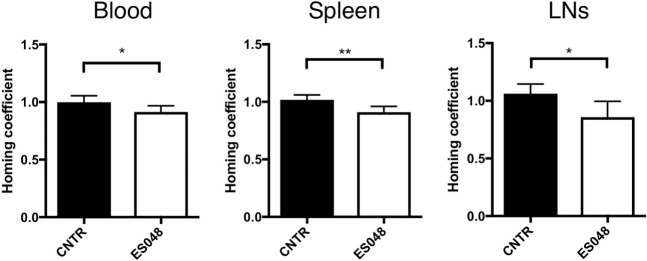
Homing of CD4^+^ T cells into secondary lymphoid tissues depends on neutral sphingomyelinase function. CD4^+^ T cells were isolated from spleens and LNs of Thy1.1^+^ donor mice, solvent or inhibitor treated, labeled, and a 1:1 ratio of labeled cells, inhibitor treated or not, was re-injected into acceptor mice. After 1 h, blood, spleen, or LN samples were isolated and analyzed for the frequency of Thy1.1^+^ cells by flow cytometry. Bars show means with SD for *n* = 6 mice. Homing coefficient: % subpopulation in inoculum/% subpopulation retrieved.

Cytoskeletal and membrane dynamics are important in T cell motility, and also known to be subject to regulation by NSM activity. To establish whether both phenomena are linked, we comparatively analyzed the steady-state homing efficiency of NSM inhibited and unmodified CD4^+^ T cells in an *in vivo* homing assay under non-inflammatory conditions. Titration experiments revealed that the inhibitor ES048 (Figure S1A in Supplementary Material) did not affect viability of CD4^+^ T cells up to a concentration of 2.5 µM. It revealed no effect on ASM activity using the recombinant enzyme (Figure S1B in Supplementary Material). When tested in splenocyte extracts, it specifically inhibited NSM activity up to a concentration of 2 µM; while at higher concentrations, ASM activity was also slightly affected (Figure S1C in Supplementary Material). Therefore, the ES048 was used at 1.5 µM further on. Using these conditions, inhibition of NSM activity persisted after removal of ES048 [70.73% after 1 h, 48.00% after 9 h, 23.11% after 16 h (Figure S2B in Supplementary Material)]. NSM ablation also did not affect the expression of CCR7 and CD62L, the receptors contributing to T cell homing (Figures S3A–D in Supplementary Material). Thy1.1^+^ CD4^+^ T cells were inhibitor or solvent treated for 2 h, labeled with eFluor 670 or CFSE, respectively. A 1:1 mixture of both populations was transferred to Thy1.2^+^ recipient mice and Thy1.1^+^ cells were recovered after 1 h. Then, homing of ES048-pre-treated Thy1.1^+^ T cells in spleen and LN was significantly lower than that of solvent-treated cells (Figure 1; ratio 1:0.89 for spleen, and 1:0.81 for LNs, middle and right panels). However, the recovery of ES048-treated cells from peripheral blood was similarly reduced as that in the spleen (ratio homing coefficient solvent- versus ES048-treated cells 1:0.91) (Figure 1, left panel). These data indicate the importance of NSM activity in rapid T cell homing to lymph nodes in an uninflamed environment, hence, in case of an immediate immune reaction where quick recruitment of effector cells is essential, this could be highly relevant for the initiation of the immune response.

### SMase Function Promotes Adhesion and Transendothelial Migration of T Cells to Human Brain Microvascular Endothelial Cells (HBMECs)

**Figure 2:**
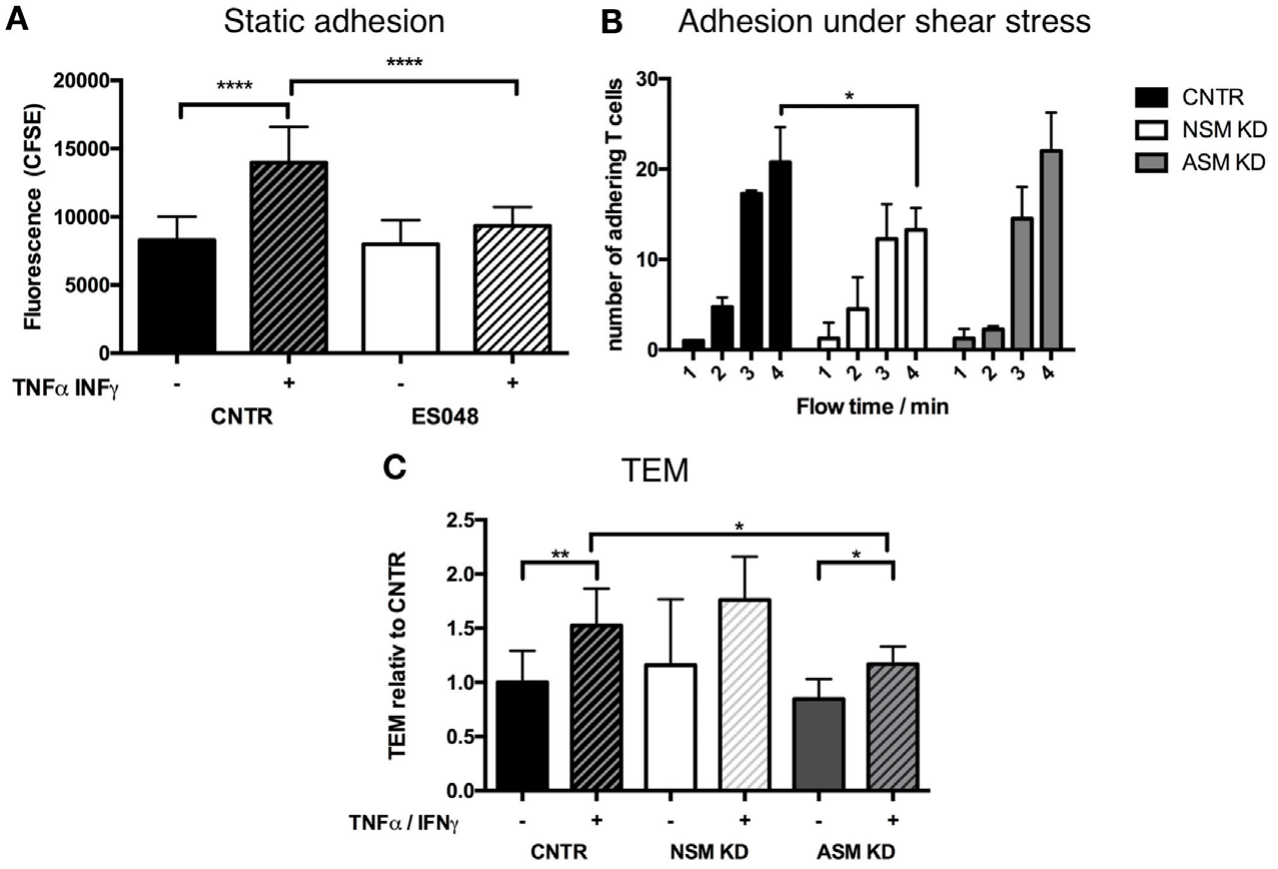
Functional sphingomyelinases promote adhesion of T cells to human brain microvascular endothelial cells (HBMECs) and TEM. **(A)** CFSE-labeled T cells (exposed to solvent or 1.5 µM ES048 for 2 h) were seeded onto resting (−) or over night TNFα/IFNγ (+) stimulated HBMECs in 96-well plates for 30 min. Following removal of non-adherent cells, cells were lysed and CFSE-fluorescence determined. Graph shows means with SD for *n* = 3. **(B)** CNTR, neutral sphingomyelinase (NSM) KD and acid sphingomyelinase (ASM) KD T cells were introduced into a flow of 11 mL/h over TNFα/IFN stimulated HBMECs. Adhering T cells were quantified after 1, 2, 3, and 4 min flow time. Bars show means with SD of *n* = 3, average knockdown efficiency: NSM: 61.67%, ASM: 82.67%. **(C)** HBMECs were seeded in filter inlets until resistance reached >60 Ω/cm^2^. HBMECs were treated as in panel **(A)**, and 100 ng/mL SDF-1α was added to the lower compartment. T cells in the upper compartment transmigrated toward SDF-1α for 1 h before counting. Percentages of transmigrated T cells were stratified for the number of T cells adhering to the HBMECs determined as in panel **(A)**. Values shown in the graph were normalized to the value for TEM in untreated HBMECs (CNTR set to 1) and are means with SD (*n* = 5), average knockdown efficiency: NSM: 81.00%, ASM: 82.40%.

To get insight into the mechanism of how the NSM might regulate T cell homing, we analyzed its role in steps shown to be important in processes, such as endothelial adhesion, endothelial transmigration (TEM), and directional movement toward a chemokine source (e.g., an inflammation) *in vitro* using primary human T cells. Though ES048 is an NSM inhibitor at the concentration used (Figures S1A–C in Supplementary Material, and see above), the specific contribution to the biological responses studied now *in vitro* were paralleled by siRNA genetic knockdown of the enzyme. This was not possible for the *in vivo* tranfer experiment because nucleofection of primary T cells generally affected T cell motility (also for the CTRL cells) (not shown). As indicated for murine CD4^+^ T cells, the inhibitor ES048 also did not interfere with the viability of human T cells and NSM inhibition was retained after removal of the inhibitor for at least 9 h (not shown). For endothelial adhesion, T cells exposed to ES048 or solvent were seeded onto confluent layers of HBMECs which were resting or had been pre-activated by an over night treatment with TNFα/IFNγ which promotes upregulation of adhesion receptors and mimics an inflammatory environment. While control and inhibitor-treated cells adhered equally well to the resting endothelium (black and white bar in Figure 2A), endothelial activation (+TNFα/IFNγ) clearly enhanced adhesion of control cells but not that of inhibitor-treated cells (Figure 2A, hatched bars). Control siRNA transfected T cells (CNTR) also showed an increased adhesion to activated HBMECs under shear stress compared to NSM KD T cells, treated with an siRNA specifically targeting the NSM (Figure 2B). These findings reveal that NSM activity is required for T cell adhesion to an inflamed, but not a resting endothelium.

To analyze the contribution of the NSM to TEM, this enzyme was genetically depleted from primary T cells by siRNA. NSM ablated (NSM KD) and control siRNA transfected T cells (CNTR) inefficiently passed through resting HBMECs, and this was enhanced for both NSM-deficient and -sufficient T cells under inflammatory conditions (Figure 2C, with only values of CNTR reaching statistical significance), indicating that NSM may not be required for TEM. As the ASM in endothelial cells was reported to be important in this process (26), we analyzed whether this enzyme would be required in T cells as well. Indeed, ASM ablation did not affect TEM under resting conditions, but reduced TEM increase under inflammatory conditions (Figure 2C). Altogether, this indicates that NSM activity is more important for adhesion to than for passage through the endothelium, while ASM activity plays an important role in the transmigration process through the HBMECs, but not for adhesion as shown for adhesion under shear stress (Figure 2B).

### NSM Function Is Required for High-Affinity LFA-1 Formation and Clustering

**Figure 3:**
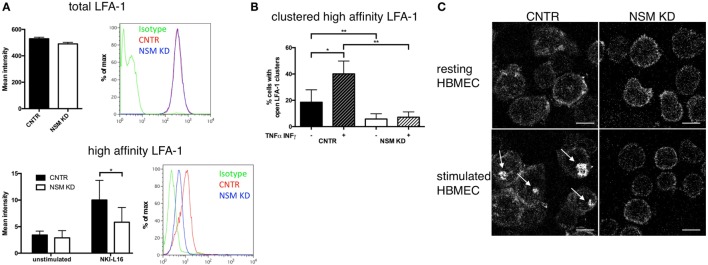
Neutral sphingomyelinase (NSM) function is required for induction and clustering of high-affinity LFA-1. **(A)** Total LFA-1 was stained on resting CNTR or NSM KD T cells with a pan LFA-1 antibody (top graph and histogram). T cells (CNTR or NSM KD) were left untreated or stimulated in solution with 100 ng/mL NKI-L16 for 30 min, before fixation and staining of high-affinity LFA-1 (bottom graph and histogram). Bars represent means with SD (*n* = 4), average knockdown efficiency: 61.50%. **(B,C)** Human brain microvascular endothelial cells (HBMECs) were left in a resting state [− **(B)** or upper row **(C)**] or stimulated with TNFα/IFNγ [+ **(B)** or bottom row **(C)**] over night before addition of control or NSM KD T cells for 30 min and staining for high-affinity LFA-1. Z-stack images were taken, and clusters were analyzed manually. Graph (left) shows means with SD for *n* = 3 experiments, average knockdown efficiency: 85.67%. Images on the right show representative cells for each condition with arrows pointing at the LFA-1 clusters (control T cells, left panel; NSM KD, right panel). Scale bars, 5 µm.

Because LFA-1 activation is critical for T cell adhesion, we analyzed whether NSM activity would impact on affinity maturation, clustering, and redistribution of this molecule. The activating NKI-L16 antibody triggered a threefold upregulation of the LFA-1 high-affinity isoform in CNTR T cells, while this increase was only twofold in NSM KD T cells, indicating that basal NSM activity was required for the outside-in signaling resulting in LFA-1 affinity maturation (Figure 3A, bottom panel), while total LFA-1 expression was unchanged (Figure 3A, top panel). Potential NSM-relating regulations of LFA-1 high-affinity clustering and polarization were analyzed in CTRL and NSM KD T cells seeded onto HBMECs. Under resting conditions, open LFA-1 clusters were present in 18.5% of CTRL T cells, however, in only about 6% of NSM KD cells (Figure 3B, black and white bars). The frequency of CTRL T cells exhibiting open LFA-1 clusters markedly increased under inflammatory conditions (Figure 3B, dark hatched bars). Though reduced by number (Figure 2A), NSM KD T cells adherent to activated HBMECs almost entirely failed to cluster the LFA-1 open conformation (Figures 3B,C) as seen for CTRL T cells (Figures 3B,C). While this manuscript was prepared for submission, Grassmé et al. showed that indeed ceramide clusters are needed for β1-integrin accumulation on endothelial cells; however, one should keep in mind that the ASM is located at the outer leaflet of the plasma membrane while the NSM works at the inner leaflet (30). Altogether, NSM activity proved to be an important regulator of both, LFA-1 affinity maturation and clustering and thereby, essentially contribute to inefficient adherence of NSM insufficient T cells. These findings, however, might indicate that the NSM is needed for overall T cell polarization.

### NSM Ablation Reduces Cell Polarity

**Figure 4:**
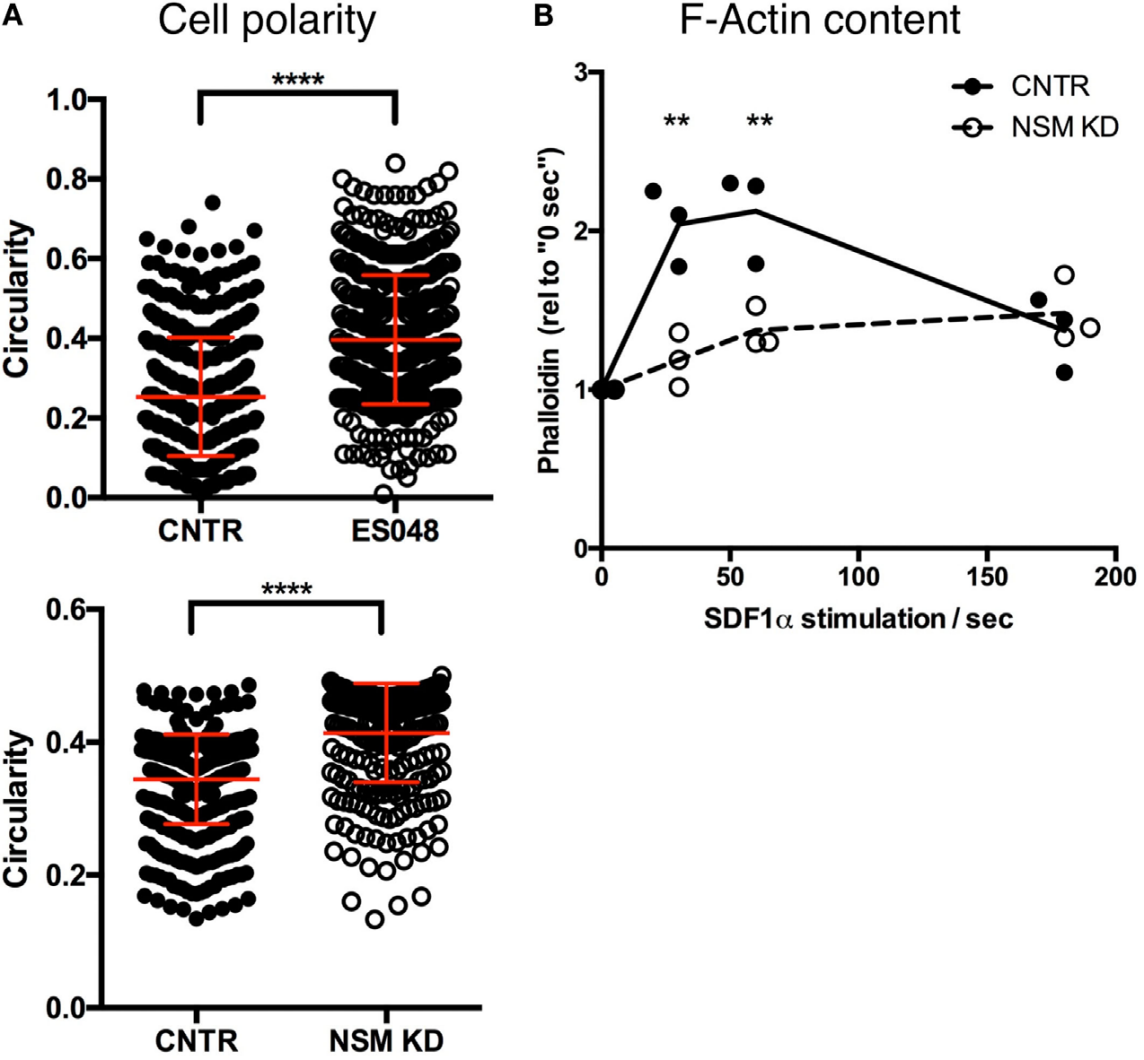
Neutral sphingomyelinase (NSM) ablation limits cell polarity. **(A)** Solvent or ES048 treated (top graph) or CNTR and NSM KD (bottom graph) CFSE-labeled T cells polarized on resting human brain microvascular endothelial cells for 2 h prior to fixation and T cells’ shape was analyzed with Fiji software. Graph shows means with SD for *n* = 3, at least 150 cells were analyzed per experiment, average knockdown efficiency: 74.00%. **(B)** Control and NSM KD T cells were stimulated with SDF-1α for 30 s, 1 and 3 min, fixed and F-actin was labeled for flow cytometric analysis. Dots represent data from individual experiments, lines the mean of *n* = 3, average knockdown efficiency: 66.00%.

To analyze NSM-relating impacts on T cell morphology, we recorded the circularity of T cells (NSM-inhibited or not, as well as NSM genetically ablated or not) seeded onto resting HBMECs after 30 min. In the absence of the inhibitor, T cells revealed a circularity value of on average 0.25, while NSM-inhibited cells mostly retained a round, circular shape [circularity value of 0.40 (Figure 4A, top graph)]. The same result was obtained using CNTR and NSM KD T cells, with a circularity value of 0.34 for CNTR and 0.41 for NSM-ablated T cells (Figure 4A, bottom graph).

Morphological polarization is usually associated with actin cytoskeleton remodeling and, therefore, transient F-actin polymerization that did occur in primary T cells after stimulation with SDF-1α as expected (Figure 4B). By stark contrast, F-actin content only slightly increased in NSM KD cells never approaching maximum levels seen in controls (Figure 4B), indicating that the NSM is required for stimulated reorganization of the actin cytoskeleton.

**Figure 5:**
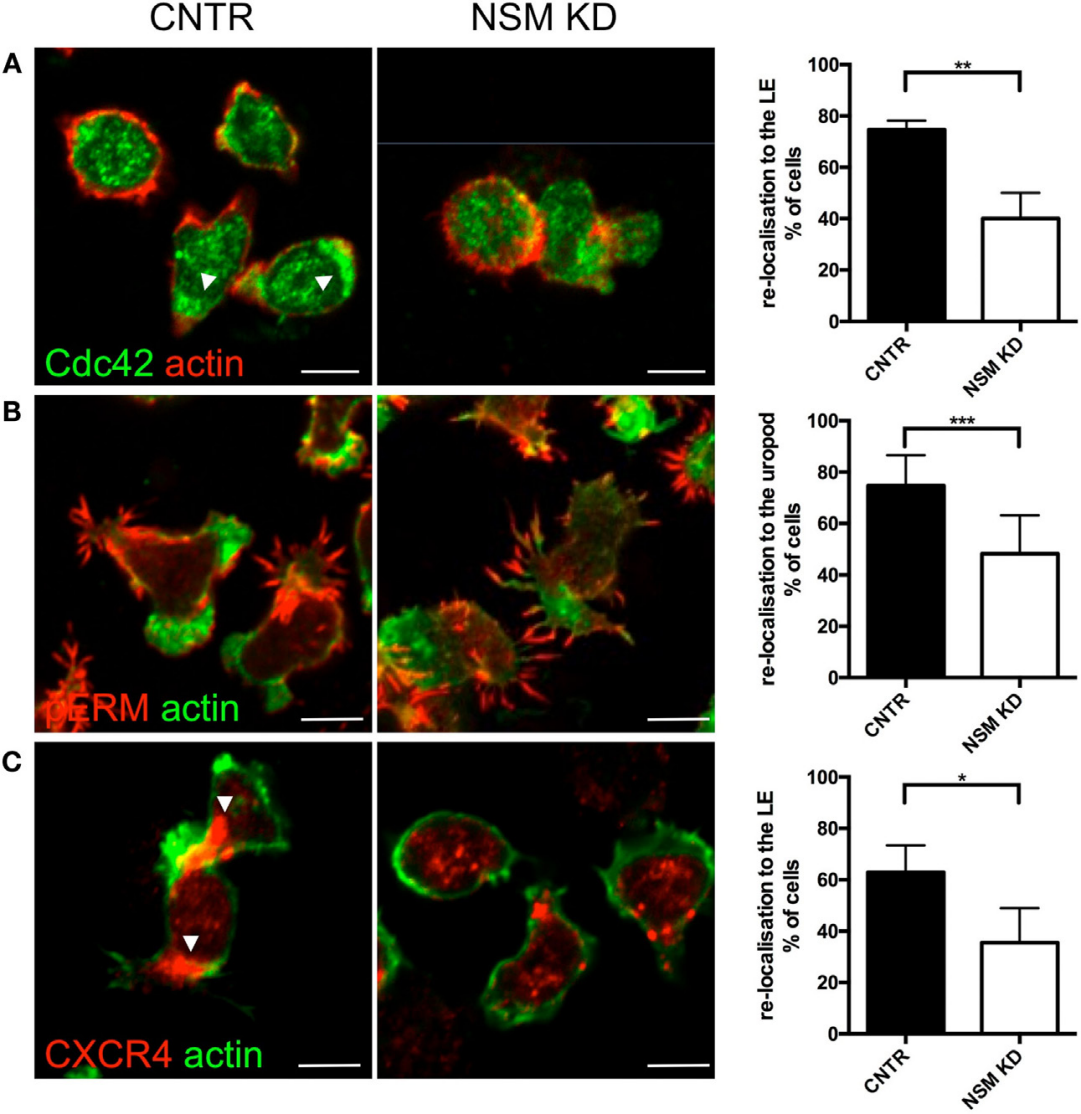
Neutral sphingomyelinase (NSM) is essential for CXCR4, pERM, and Cdc42 polarization of T cells on FN. Primary control and NSM KD T cells were stimulated with 100 ng/mL SDF-1α, settled onto FN-coated slides for 1 h, stained for Cdc42 **(A)**, pERM **(B)**, and CXCR4 **(C)**, and redistribution toward the leading edge (LE) or uropod, as indicated in the graphs, was analyzed manually. Arrowheads mark CXCR4 or Cdc42 accumulations. Images show representative cells, the graphs (right) show quantification of re-localization with means and SD for *n* = 3 independent experiments with approximately 30 cells each, average knockdown efficiency: 85.67%. Scale bars, 5 µm.

To evaluate whether the inability of NSM-deficient T cells to polarize would extend to intracellular regulators of the cytoskeletal machinery, subcellular redistribution of Cdc42 and pERM was analyzed in NSM KD and CTRL T cells migrating on FN coated slides. In CNTR T cells Cdc42 localized to the LE of the polarized T cells, while this was much less pronounced for NSM KD cells where the molecule tended to accumulate in spot-like structures at various cell membrane areas (Figure 5A, top row, arrowheads; polarization index: 75% for CRTL, 39% for NSM KD cells). While expectedly 85% of CTRL cells polarized pERM within protrusions of the uropod, this subcellular distribution was confined to 58% of NSM KD cells, which tended to form pERM^+^ extensions at multiple sites of the cells (Figure 5B). These data indicate that NSM activity is required for a step prior to the activity of proteins regulating actin dynamic such as, Cdc42 and ERM proteins, and affects overall cell polarity induced by polarizing signals.

We, therefore, analyzed whether the latter can be provided in NSM-deficient cells and analyzed polarized redistribution of CXCR4 as important in perceiving and relaying SDF-1α signaling. As seen previously for open LFA-1 (Figure 3C), CTRL cells, but not NSM KD cells efficiently polarized CXCR4 toward their LE (65 versus 40%, Figure 5C) indicating that signals received through this receptor are not spatially confined and thereby, though relayed [as NSM ablation did not ablate CXCR4 expression (Figure S4 in Supplementary Material)] cannot be translated into a spatially meaningful cytoskeletal response.

### Directional T Cell Migration in a Confined Environment Requires NSM Activity

**Figure 6:**
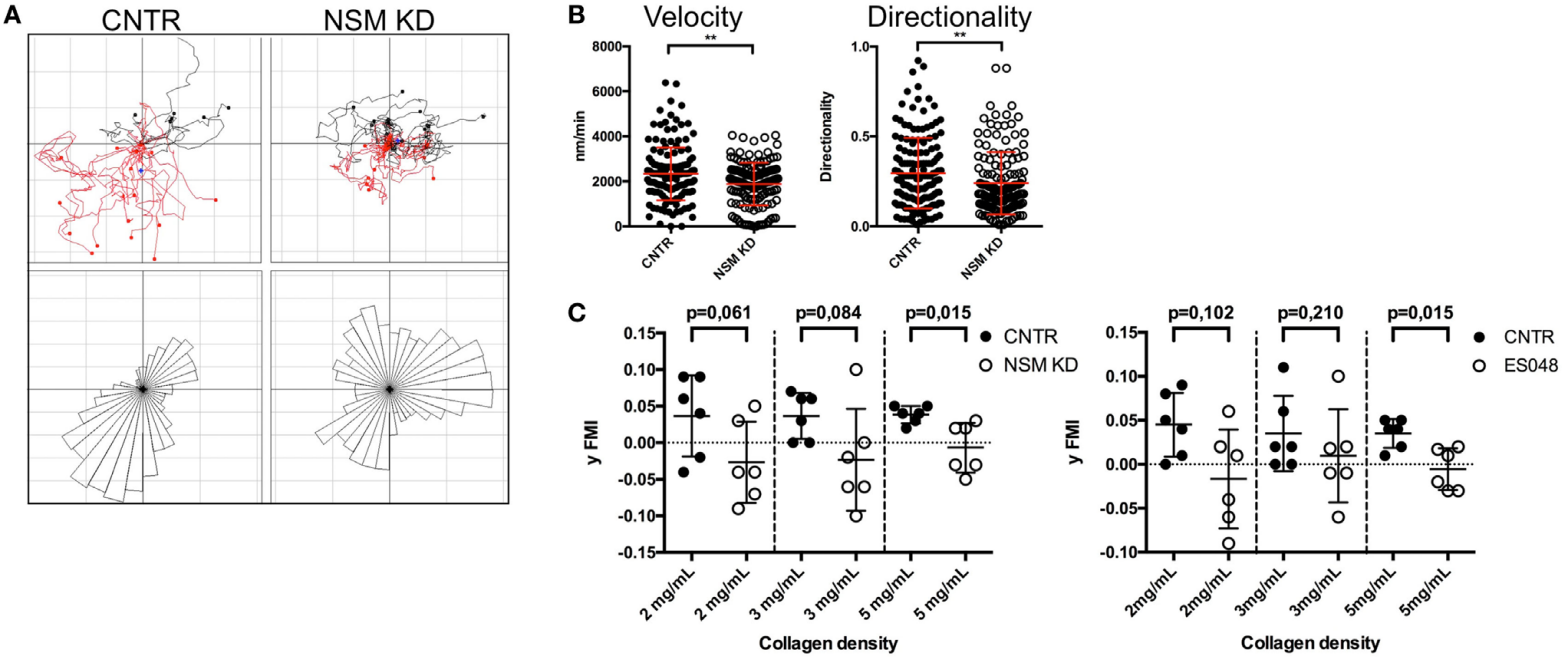
Neutral sphingomyelinase (NSM) function is crucial for directed T cell migration in a confined environment. **(A)** Individual tracks of control (upper left) or NSM KD T cells (upper right) embedded in 3D collagen (4 mg/mL) matrices are depicted in the coordinate system. SDF-1α was applied to one side of the channel (bottom in the images), and cells were imaged for 90 min. The bottom panels show rose diagrams visualizing the directional movement. **(B)** Velocity (left) and directionality (right) of cells from panel **(A)**. Graphs show means with SD for *n* = 4 with at least 30 cells per experiment, average knockdown efficiency: 68.00%. **(C)** CNTR and NSM KD (left graph) or control or ES048-treated (right graph) T cells were embedded in collagen matrices with densities of 2, 3, or 5 mg/mL and tracked for 90 min. The forward migration index in *y*-direction (y FMI) was calculated with Fiji software; each dot represents one experiment with at least 30 cells. Graph shows means with SD for *n* = 3, average knockdown efficiency: 77.00%.

In response to chemotactic signals such as provided through CXCR4, cytoskeletal reorganization is essential for directional T cell migration. The potential impact of the NSM in this process was analyzed in 3D environments using collagen type I matrices. As migration on collagen is not β_2_ integrin dependent, this system allows for an analysis of direction sensing and cytoskeletal rearrangements in T cells without the impact of defective adhesion measured for NSM-deficient cells (Figures 2A,B). The movement of CNTR and NSM KD T cells along a gradient of SDF1-α was tracked over 90 min. The movement of CNTR T cells covered an overall larger area than that of NSM KD cells (Figure 6A, tracks in upper panel), and was more directional toward the chemokine source (Figure 6A, rose diagrams in the bottom panel). While 69.6% of control cells efficiently followed the gradient, this was significantly impaired in NSM KD T cells (only 46.1%). On a quantitative basis, directionality values were 0.30 for CNTR and 0.24 for NSM-ablated T cells (Figure 6B, right graph). In addition, the movement velocity was slightly reduced for NSM KD T cells (2.3 µm/min for CNTR and 1.9 µm/min for NSM KD T cells; Figure 6B, left graph), indicating that the NSM might not only control the directionality but also the speed of T cell migration. Hypothesizing that the impact of the NSM in this process might be more pronounced in restrictive environments, we varied the density of the collagen meshwork and analyzed the forward migration index in *y*-direction of T cells which represents the efficiency of the forward migration of cells. In a looser network (2 mg/mL collagen), NSM inhibition did not affect the range of T cell movement (Figure 6C; left graph for knockdown and right graph for inhibitor treatment). With increasing density of the network (3 and 5 mg/mL collagen), the range of movement of both, control and NSM-ablated cells, was significantly impaired, while the impaired sensing of the gradient in NSM-ablated cells showed at all collagen concentrations. The analysis of the 3D migration clearly shows an important impact of a functional NSM on the migration capacity of primary T cells, which is most obvious when the environment is restrictive, and, therefore, extensive cytoskeletal and membrane deformations are needed.

## Discussion

Sphingolipids are major components of the plasma membrane and, therefore, the activity of enzymes regulating their homeostatic composition and their dynamic alteration upon cellular activation are of fundamental importance for dynamics and biophysical properties of the membrane itself, sorting of integral membrane proteins and membrane proximal signaling complexes, as well as membrane coupling to the cytoskeleton (31). These processes are particularly important in migrating cells such as T lymphocytes where morphological and functional polarization is a prerequisite for efficient directional chemotactic responses, for endothelial passage and homing to secondary lymphatic and peripheral tissues (32). We now show that the NSM that hydrolyzes sphingomyelin at cytosolic membrane leaflets contributes to directional T cell migration *in vitro* and *in vivo*, with cellular polarization, adhesion, and actin reorganization being major regulated targets.

The importance of the NSM in T cell homing became especially important early following transfer of inhibitor-treated cells that were compromised in LN homing under steady-state conditions (Figure 1). Because nucleofection of primary T cells generally affected T cell motility (also for the CTRL cells), genetic ablation of the enzyme could not be performed for the *in vivo* assay (not shown). NSM-related differences in steady-state T cell homing were particularly pronounced in the LN where, in contrast to the spleen, access is restricted by the endothelial barrier (HEV) (33, 34). Our flow cytometry-based analysis of LN accumulation of bulk CD4^+^ T cells does, however, not allow to address a potential differential effect of NSM ablation on particular T cell subsets or if, in addition to entry, intra-LN trafficking to T cell compartments is also subject to NSM regulation. *In vivo*, the absence of NSM activity appeared to delay, but not preclude homing as NSM-ablated T cells efficiently accumulated 16 h following transfer in all compartments analyzed (Figure 1). We can, however, not exclude that the NSM inhibition by ES048 *in vivo* is less stable than measured *in vitro* (Figure S2B in Supplementary Material) and, therefore, the enzyme activity may have already recovered after 16 h. Because *in vitro*, the importance of the NSM on polarization and adhesion to the inflamed endothelium was much more pronounced than for resting conditions, it is very likely that the role of the enzyme *in vivo* would be even more prominent upon inflammatory conditions.

Morphological and functional polarization proved to be major targets of NSM activity *in vitro* (Figures 4 and 5). At the level of functionally relevant surface receptors, this was particularly evident for CXCR4 and LFA-1 which—though expressed at levels equivalent to those of CTRL cells (Figure 3A; Figure S4 in Supplementary Material)—were inefficiently redistributed upon exogenous stimulation by the respective chemokine, fibronectin, or endothelial cells (Figures 3B and 5C). A direct role of the NSM in signaling did not become evident for either of the two receptors (not shown) and, therefore, the failure of polarized sorting and, for LFA-1 clustering, rather than interference with receptor signaling is likely to account for the functional impairment measured in the T cells.

Cholesterol has recently been shown to directly modulate chemokine receptor function (35) and, therefore, organization of steady-state membrane domains enriched in ceramide might influence receptor organization. Association of CXCR4 with lipid rafts also was reported to be of functional importance (36, 37), but did, however, not detectably differ between NSM KD and CTRL cells (not shown). Therefore, the NSM-regulated polarized redistribution might be particularly important for spatial integration and translation of signals into directional migration motility in 2D and especially 3D environments (Figure 6) (38). As the role of the NSM became more prominent in complex environments (Figure 6C), it is likely that NSM-dependent regulation of actin cytoskeletal dynamics independent of CXCR4 signaling efficiently contributes to directional motility in tissues. This is further evidenced by the formation of extensive protrusions over the entire cell body rather than polarized, and lamellipodial extensions, as revealed by F-actin detection (Figure 5B). Similar observation with regard to formation of non-directional pseudopodia have been previously reported for leukocytes from FAN-deficient zebrafish embryos (39). FAN couples TNF-R signaling to NSM, however, a role of this enzyme in pseudopodia formation in the zebrafish system studied has not been directly addressed. Its involvement there is, however, quite possible because NSM has been shown to be important in directional pseudopodia formation in fMLP-stimulated neutrophils earlier (29).

Similar as for CXCR4, LFA-1 function was found sensitive to the plasma membrane lipid-nano-environment with ceramide content being especially important for integrin mobility and ligand encounter (40). Our findings that both surface levels of high-affinity (but not total, Figure 3A) LFA-1 and their clustering upon adherence to inflamed HBMECs were substantially reduced on the surface of NSM KD T cells indicate a requirement of the NSM for inside-out signaling for LFA-1 activation, and thereby, largely explains reduced adhesion to the stimulated HBMECs (Figures 2A,B). The role of the NSM in LFA-1 activation is unknown, but may involve talin that acts as an LFA-1 linker to the actin cytoskeleton and promotes unfolding of bent LFA-1 and exposition of its ligand binding domain and thereby, acquisition of the open confirmation (41, 42). In contrast to other cytoskeletal adaptors such as the ERM proteins (43), regulation of talin activity by lipid membrane domain organization is unknown and its analysis is beyond the scope of our present study.

In contrast to polarization and adhesion, NSM activity was not required for TEM which was rather dependent on ASM activity thereby revealing that the enzymes have an important, non-redundant role in endothelia interactions (Figure 2C). Interestingly, ASM activity in endothelial cells was also required for efficient transmigration of T cells (26), where the role of NSM has not been studied as yet. The differential impact of the NSM and the ASM on adhesion and transmigration in T cells may be due to the different cytoskeletal mechanisms involved (44, 45). Because migratory T cells convert their lamellipodia/uropod-based phenotype into a blebbing/transmigratory phenotype by microtubule depolymerization and increased RhoA/ROCK activity (46), the NSM [important for the organization of both the lamellipodium and the uropod (Figure 5)] can impact on both migration and adhesion. Transmigration requires a pushing force to scan the endothelium for suitable TEM spots (47), formation of invasive podosomes (45), and uropod contraction that relies on RhoA activation (48, 49), which has been reported as a downstream effector of ASM activation and ceramide release (50).

At a molecular level, the NSM appeared to be a key regulator of actin cytoskeletal dynamics and functional cell polarization in T cells in our study. NSM-dependent physiological regulation of actin dynamics in TCR/CD28 co-stimulated T cells has been reported as well as actin cytoskeletal breakdown upon exaggerated NSM activation (28). In this regard, lack of redistribution of central regulators of actin polymerization and organization, such as Cdc42 and pERM, in the absence of NSM are not likely to be the cause, but rather consequence of impaired cell polarization (which they can certainly amplify) (Figures 5A,B). At a functional level, the importance of NSM-dependent redistribution of Rac and RhoA has been linked to regulation of directional motility of neutrophils (29). In this study, loss of directional motility upon pharmacological NSM inhibition was partially restored upon exogenous supply of ceramides. Because NSM releases ceramides at the inner leaflet of the plasma membrane and the flipping dependent exchange of complex sphingolipids between membrane leaflets is slow, it is unclear to what extent these externally fed ceramides accumulate in the inner leaflet of the plasma membrane to substantial amounts. Therefore, the mechanism underlying this recovery remains unclear.

In line with our finding of cell polarity being a major NSM target in T cells, a role of this enzyme in this process has been evidenced upon its pharmacological inhibition in differentiating neuronal cells where, *via* activation of atypical PKC, NSM and ceramides were involved in stabilization of primary cilia (51). Though the NSM has not been specifically addressed in this study, ceramide accumulation has also been found crucial for acquisition of cell polarity in primary ectoderm cells where it controlled polarized sorting of Cdc42, again *via* activation of atypical PKCs (52). It is, therefore, quite possible that downstream effector of NSM to be identified in T cells also control cell polarization in response to chemokine receptor signaling, β1 integrin ligation, and contact with activated endothelial cells.

## Materials and Methods

### Ethics Statement

Primary human cells were obtained from the Department of Transfusion Medicine, University of Würzburg. All experiments involving human material were conducted according to the principles expressed in the Declaration of Helsinki and ethically approved by the ethical committee of the Medical Faculty of the University of Würzburg. C57BL/6 and Thy1.1 congenic C57BL/6 mice were bred under specific pathogen-free conditions in the animal facility of the Institute for Virology and Immunobiology, Würzburg. All animal experiments were conducted in accordance with German law and approved by the Government of Unterfranken.

### Murine CD4^+^ T Cell Isolation and Homing Assays

LNs and spleen were isolated from Thy1.1 congenic C57BL/6 mice, CD4^+^ T cells were isolated by magnetic sorting (MagniSort Mouse CD4 T Cell Enrichment Kit, affymetrix/eBioscience, San Diego, CA, USA), and divided into two populations, one of which was incubated with EtOH (1:1,000, 2 h) and labeled with eFluor 670 (affymetrix/eBioscience, San Diego, CA, USA; 5 µM, 10 min), and the other treated with ES048 (2 h, 1.5 µM) and subsequently CFSE labeled (affymetrix/eBioscience, San Diego, CA, USA; 5 µM, 10 min). To exclude an impact of the dye, labels were switched for half of the transfers. T cells were mixed at a 1:1 ratio and a total of 1 × 10^7^ cells of the mixture was injected into the tail vain of C57BL/6 mice. After the time periods indicated, T cells were harvested from the blood, LN, and spleen and the frequency of Thy1.1^+^/dye positive cells was analyzed by flow cytometry.

### Cells and Nucleofection

Primary human PBMCs were isolated from peripheral blood obtained from healthy donors by Ficoll gradient centrifugation. CD3^+^ T cells (purity ~90%) were enriched using nylon wool columns and maintained in RPMI1640/10% FCS (Gibco/Thermo Fischer, Waltham, MA, USA and Biochrom AG, Berlin, Germany). T cells [when indicated pre-exposed to ES048 (2 h, 1.5 µM)] were transferred onto human brain microvascular endothelial cells (HBMEC, kindly provided by J. Schneider-Schaulies) cultured in 96-well plates (for adhesion and polarization assays) or into collagen matrices (for 3D migration assays). HBMECs were cultured in Endothelial Cell Growth Medium MV (PromoCell, Heidelberg, Germany), seeded into 96-well plates or 3.0-µm pore polycarbonate membrane transwell inserts (Corning, Kennebunk, ME, USA) and stimulated with 100 U/mL TNFα and 500 U/mL IFNγ (both Miltenyi Biotec, Bergisch Gladbach, Germany) over night when indicated. Nucleofection of primary human T cells was performed according to the manufacturer’s protocol (Amaxa). For NSM2 or ASM silencing, human T cells were nucleofected twice at a 2-day interval with 100 pmol siRNA targeting human SMPD3 (NSM2) (5′-UGCUACUGGCUGGUGGACC-3′, 5′-GGCUCCACCAGCCAGUAGCA-3′), human ASM (5′-CCAUGAAAGCACACCCGUC-3′, 5′-GACAGGUGUGCUUUCAUGG-3′) or, for control, a non-targeting siRNA (Sigma-Aldrich, St. Louis, MI, USA). Cell aliquots were harvested at day 5 for activity assays.

### SMase Activity Assay

Acid sphingomyelinase or NSM activities were determined as previously described (28). 1 × 10^6^ T cells were disrupted by freeze/thawing in ASM or NSM lysis buffer (20 mM HEPES, 2 mM EDTA, 5 mM EGTA, 5 mM DTT, 1 mM Na-orto-vanadate, 10 mM β-glycerolphosphate, pH 5.2 for ASM, and pH 7.4 for NSM). Nuclei were removed by centrifugation for 1 min at 3,400 × *g*. Supernatants were incubated with 1.35 mM HMU-PC (6-hexadecanoylamino-4-methylumbelliferyl-phosphorylcholine) (Moscerdam substrates, Oegstgeest, Netherlands) as an artificial SMase substrate at 37°C for 17 h (final volume 30 µL). Fluorescence reading was performed using excitation at 404 nm and emission at 460 nm according to the manufacturer’s protocol. On average, knockdown efficiencies were higher than 75% at enzyme activity level (Figure S2A in Supplementary Material).

For detection of SMase activities in splenocytes, 1 × 10^6^ freshly prepared cells were treated with indicated concentrations of ES048 for 2 h at 37°C. After completion of the incubation, cells were centrifuged and the cell pellets were shock frozen in liquid nitrogen. For NSM activity, cells were resuspended in 200 µL NSM assay buffer consisting of 100 mM HEPES (pH 7.4), 5 mM MgCl_2_, 0.2% NP40, and 10 µg/mL each of aprotinin and leupeptin and sonicated for 10 s using a tip sonicator. 150 µL of cell lysate was used for the assay. For ASM activity measurement, cells were lysed for 10 min in 200 µL ASM lysis buffer containing 250 mM sodium acetate (pH 5.0), 1% NP40 and 100 µM zinc chloride. 20 µL of the cell lysate were applied to the reaction mix. 100 pmol of BODIPY-labeled sphingomyelin (Invitrogen), suspended in NSM assay buffer or ASM assay buffer (250 mM sodium acetate, pH 5.0, 0.1% NP40, and 100 µM zinc chloride), respectively, were added to start the reaction at 37°C for 1 h (300 rpm). The reaction was terminated by lipid extraction with CHCl_3_:CH_3_OH (2:1, v/v). Phases were separated by centrifugation and the organic phase was collected and vacuum-dried. Lipids were dissolved in CHCl_3_:CH_3_OH (2:1, v/v) and spotted onto a thin layer chromatography (TLC) plate. Separation of the lipids on the TLC plates was achieved with CHCl_3_:CH_3_OH (80:20, v/v). The TLC plates were analyzed using a Typhoon FLA 9500 (GE) and quantified with ImageQuant software (GE Healthcare).

### ASM Activity Assays Using Recombinant Human Acid Sphingomyelinase (rec hASM)

From a small collection of newly synthesized compounds, the inhibitor ES048 (Figure S1A in Supplementary Material) was identified to inhibit NSM at an IC50 ~2.5 μM; while at this concentration, no inhibition of ASM was observed (Figure S1B in Supplementary Material). Full experimental details on the synthesis and characterization of ES048 will be reported in due course. rec hASM was expressed from Sf9 insect cells and purified to homogeneity, similar as described previously (53). The activity assays using rec hASM were performed as previously described (54). In a black non-binding clear bottom 96-well plate (Greiner Bio-One), the fluorescence of an ASM FRET probe (1 µM, Ex 355 nm/Em 540 nm) was measured w/o enzyme or with 0.2 µg/mL rec hASM in the presence of 0, 1.5, 3, 6, or 12 µM ES048 or in the presence of 0.2 µM TP135 (~IC50) after 30 min incubation at 37°C. All experiments were done in triplicate. Error bars indicate SD. Significance was calculated using a two-tailed *t*-test.

### Adhesion and Transmigration Assay

CFSE-labeled T cells were added to 96-well plates with a confluent HBMEC cell layer, after 30 min non-adherent T cells were removed and cells were lysed. The CFSE intensity measured by a fluorescence reader corresponded to the number of adhered T cells. For transmigration assays, 3.3 × 10^4^ HBMECs were seeded into polycarbonate membrane transwell inserts (3.0-µm pore size, Corning, Kennebunk, ME, USA), cell layer integrity was analyzed by TEER value measurement after 1 week. 1 × 10^6^ T cells were added to the upper well, 100 ng/mL SDF-1α (PeproTech, Rocky Hill, NJ, USA) to the lower well and transmigrated cells were counted with counting beads (Polysciences, Warrington, PA, USA) after 1 h. For stratification of migration to adhesion, adhering T cells and HBMECs in the insert were detached and T cells were counted with counting beads by flow cytometry. The number of transmigrated T cells was normalized to the adhered T cells in order to minimize the effects of adhesion on the transmigration data.

### Adhesion under Flow

Human brain microvascular endothelial cells were grown to confluence on gelatine-coated μ-Slides VI^0.4^ (ibidi, Munich, Germany) and stimulated with TNFα (100 U/mL) and IFNγ (500 U/mL, both: Miltenyi Biotec, Bergisch Gladbach, Germany) over night when indicated. 5 × 10^5^ CFSE-labeled control, NSM KD and ASM KD T cells in RPMI/10% FCS/1 μM HEPES (pH 7.4) were allowed to flow through the channel (flow rate: 11 mL/h, shear stress: 0.4 dyn/cm^2^ when assuming a dynamical viscosity of water at 22°C of η = 0.01 dyn × s/cm^2^). Images were taken after 1, 2, 3, and 4 min. Firmly adherent T cells were manually counted using Fiji software.

### 3D Migration Assay

For tracking experiments, 1 × 10^6^ T cells (pre-treated or not as indicated) were resuspended in RPMI/10% FCS. For collagen matrix generation, collagen I Rat Tail High Protein (Corning, Amsterdam, Netherlands) was adjusted to neutral pH with 7% bicarbonate and T cells were added on ice. Un-polymerized gels were given into μ-Slide VI^0.4^ (ibidi, Munich, Germany) and polymerization was induced at 37°C. For gradient generation, 50 µL RPMI/100 ng/mL SDF-1α were added at one side. Manual cell tracking was performed with the Fiji plug in “Manual Tracking” and the following analysis with the Fiji plug-in “Chemotaxis Tool.”

### F-Actin Polymerization Assay

5 × 10^5^ T cells in RPMI/0.5% BSA were incubated with 100 ng/mLSDF-1α, fixed with 4% cold formaldehyde, permeabilized (0.1% Triton X-100), and F-actin was stained with Actistain 488 (Cytoskeleton, Denver, CO, USA) for 1 h on ice, followed by flow cytometry.

### T Cell Polarization Analysis on HBMECs and FN

For morphological polarization analysis, 5 × 10^5^ CFSE-labeled T cells adhering to 1 × 10^5^ HBMECs in 8-well ibidi slides (ibidi, Munich, Germany) were fixed in formaldehyde (4% in PBS), imaged and T cell shape was analyzed with the Fiji plug in “particle analysis.” According to the software used, circularity is calculated with the formula “circularity = 4pi (area/perimeter^2^),” where a value of 1.0 indicates a perfect circle and values approaching 0.0 indicate an increasingly elongated polygon (see representative cells in Figure S5 in Supplementary Material).

Re-localization of pERM, Cdc42, and CXCR4 was analyzed in 5 × 10^5^ T cells seeded onto fibronectin-coated slides (8-well ibidi slides coated with 20 µg/mL fibronectin in PBS at 37°C, 2 h, Prospec, Ness Ziona, Israel) in RPMI/0.5% BSA with 100 ng/mL SDF-1α. For immunostaining, cells were fixed in formaldehyde (4% in PBS), permeabilized (0.1% Triton X-100) and stained for αCdc42 (clone P1), αCXCR4 (clone 12G5) (both Santa Cruz Biotech, Dallas, TX, USA) and αpERM (clone 41 A3, Cell Signaling, Danvers, MA, USA). Secondary antibodies for immunofluorescence used were Alexa 568 goat-α-rabbit, Alexa 488 goat-α-mouse, and Alexa 568 goat-α-mouse (all Life technologies, Carlsbad, CA, USA).

High-affinity LFA-1 was detected on primary T cells adhering to HBMECs in 8-well ibidi slides coated with gelatine. For immunostaining, cells were fixed in formaldehyde (4% in PBS) and permeabilized (0.1% Triton X-100). For high-affinity LFA-1 cluster analysis [Alexa Fluor 488 αCD11a/CD18 (clone m24, Biolegend, San Diego, CA, USA)] z-stack images were taken and clusters were analyzed in the contact plane of T cells and HBMECs as seen in the bright field image (Figure S6 in Supplementary Material). Clusters were defined as areas of at least 1 µm in diameter, showing a higher intensity than the background staining in a randomly selected area of interest in the cell. The clusters were counted manually.

In addition, F-actin was labeled using Actistain 488 (Cytoskeleton, Denver, CO, USA). Fluorochrome G (Southern Biotech, Eching, Germany) mounted samples were analyzed by confocal laser scanning microscopy using a LSM 780 (Zeiss, Germany), equipped with an incubation system and a 403 Plan-Apochromat oil objective (NA 1.4) and laser lines 488 and 633. Images were processed using confocal laser scanning microscopy software ZEN2012.

For the analysis of molecule localization, the LE and the uropod were defined as areas of maximum and minimum actin staining intensities, respectively, as measured in several areas of interests on the cell membrane. In case of multiple large lamella in one cells, their broad localization in the cell was defined as the leading front and the uropod as the area located opposite. Cells without a clear actin distribution were quantified as “not redistributed.”

### Flow Cytometry Antibodies

For murine cell analysis, αCD4-brilliant violet 421 (clone GK1.5, BD Biosciences, Franklin Lakes, NJ, USA), αCCR7-APC (clone: 4B12), αThy1.1-PE (clone HIS51) (both eBioscience, San Diego, CA, USA), αCD4-PE (clone: GK1.5), αCD62L-FITC (clone: MEL-14) (both Biolegend, San Diego, CA, USA), and Viability Dye (APC Cy7) (Thermo Fischer, Waltham, MA, USA) were used. Human cells were stained using Alexa Fluor 488 αCD11a/CD18 (clone m24), Alexa Fluor 647 αCD11a (clone HI111), Alexa Fluor 488 αCD197 (CCR7, clone G043H7), and APC αCD62L (clone: DREG-56) specific antibodies (all Biolegend, San Diego, CA, USA). Cells were incubated with the antibodies diluted in 50 µL of PBS containing 0.1% BSA and 0.02% NaN_3_ at 4°C for 30 min and analyzed by flow cytometry after one washing step. For induction of the high-affinity conformation of LFA-1, an αLFA-1 α-chain antibody (NKI-L16, 1 µg/mL, kind gift from IMJ Reinieren-Beeren and Carl Figdor, Nijmegen, Netherlands) was used.

### Statistical Analysis

Overall, data shown were acquired in at least three independent experiments involving individual donors. Data sets were analyzed for Gaussian distribution with the D’Agostino–Pearson omnibus normality test. For normally distributed data, a two-tailed Student’s *t* test was performed. If no normal distribution was present, the data was evaluated with the Mann–Whitney test. For statistical analysis of data sets (*p ≤ 0.05, **p ≤ 0.001, and ***p ≤ 0.0001) was used throughout the manuscript. Bars show means with SDs.

## Ethics Statement

This study (use of primary human T cells) was carried out in accordance with the recommendations of “name of guidelines, name of committee”; with written informed consent from all subjects. All subjects gave written informed consent in accordance with the Declaration of Helsinki. The protocol was approved by the “Ethical Committee Medical Faculty University of Wuerzburg.” Mouse experiments: this study was carried out in accordance with the recommendations of “name of guidelines, name of committee.” The protocol was approved by the “Government of Unterfranken, Germany.”

## Author Contributions

LC, EA, TW, KB-F, and NB performed the experiments. CA and ES synthesized, functionally characterized, and contributed the NSM inhibitor. LC, NB, EA, and SS-S designed and interpreted the experiments. LC, EA, and SS-S wrote the manuscript.

## Conflict of Interest Statement

The authors declare that the research was conducted in the absence of any commercial or financial relationships that could be construed as a potential conflict of interest.
