# Supplementary figures and images for: The Activity of the Neutral Sphingomyelinase Is Important in T Cell Recruitment and Directional Migration
Source: Front Immunol. 2017 Aug 21;8:1007. doi: 10.3389/fimmu.2017.01007 (PMC5566967; doi:10.3389/fimmu.2017.01007)

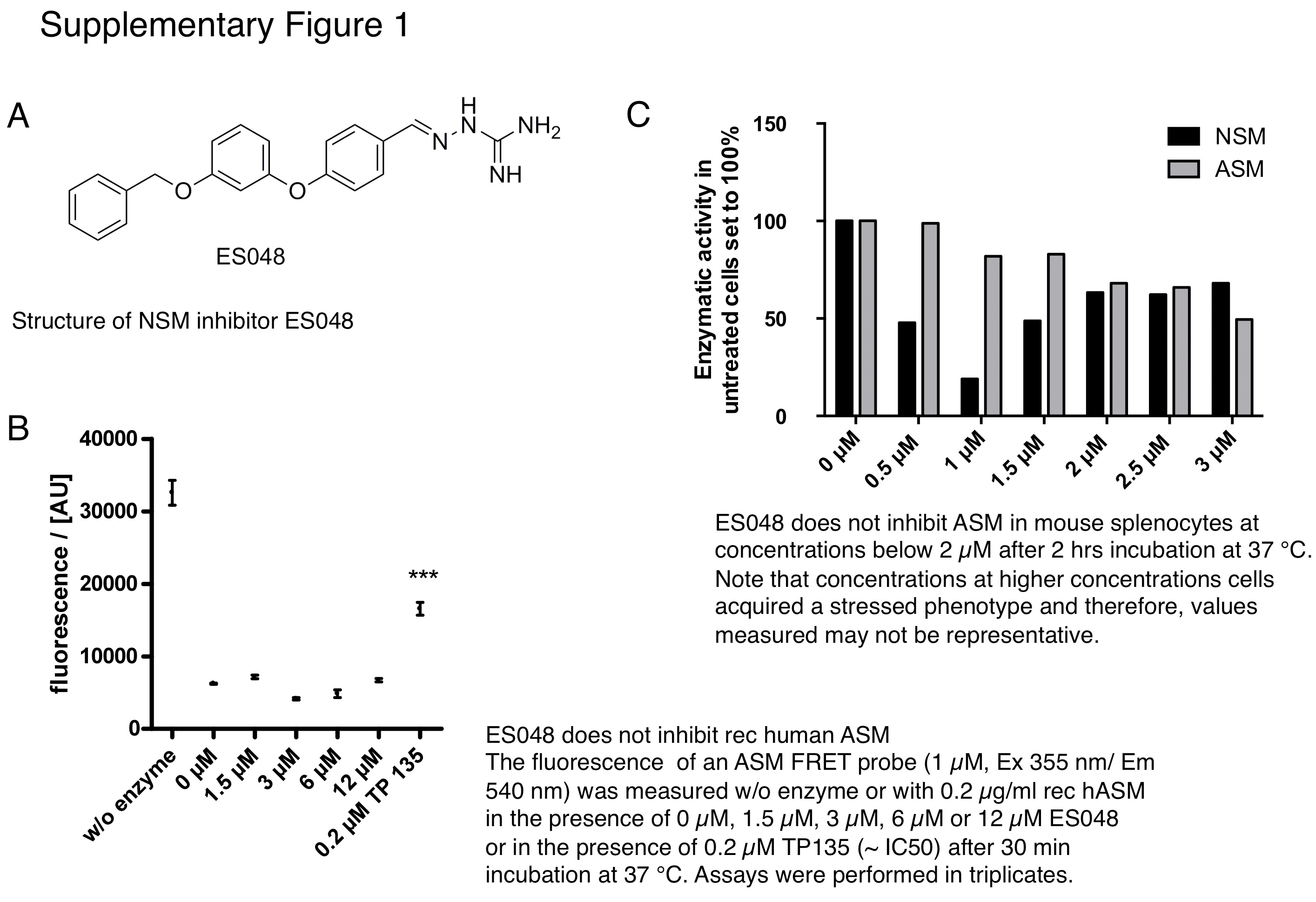

Supplement: Supplementary file 1 [file Image_1.TIF]

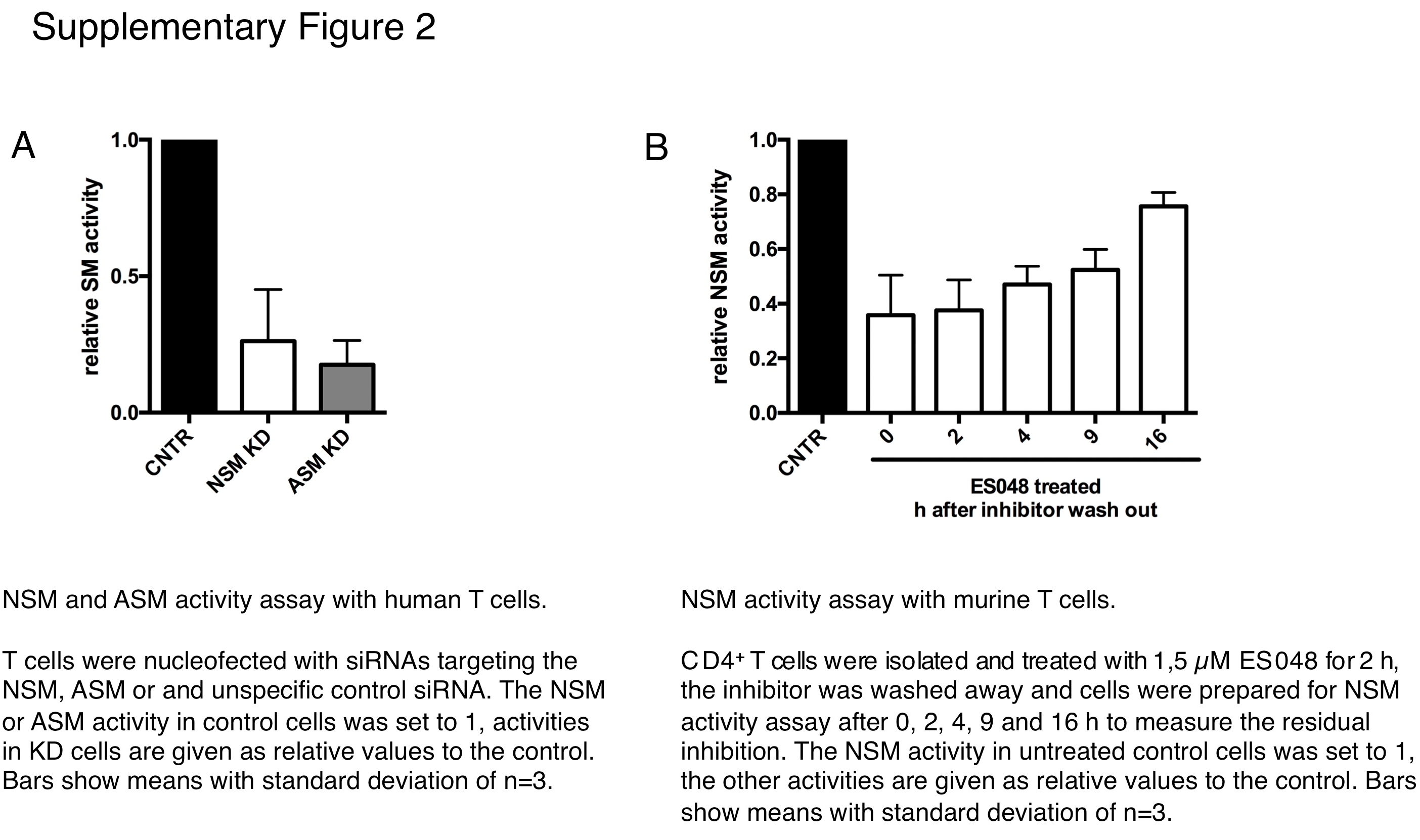

Supplement: Supplementary file 2 [file Image_2.TIF]

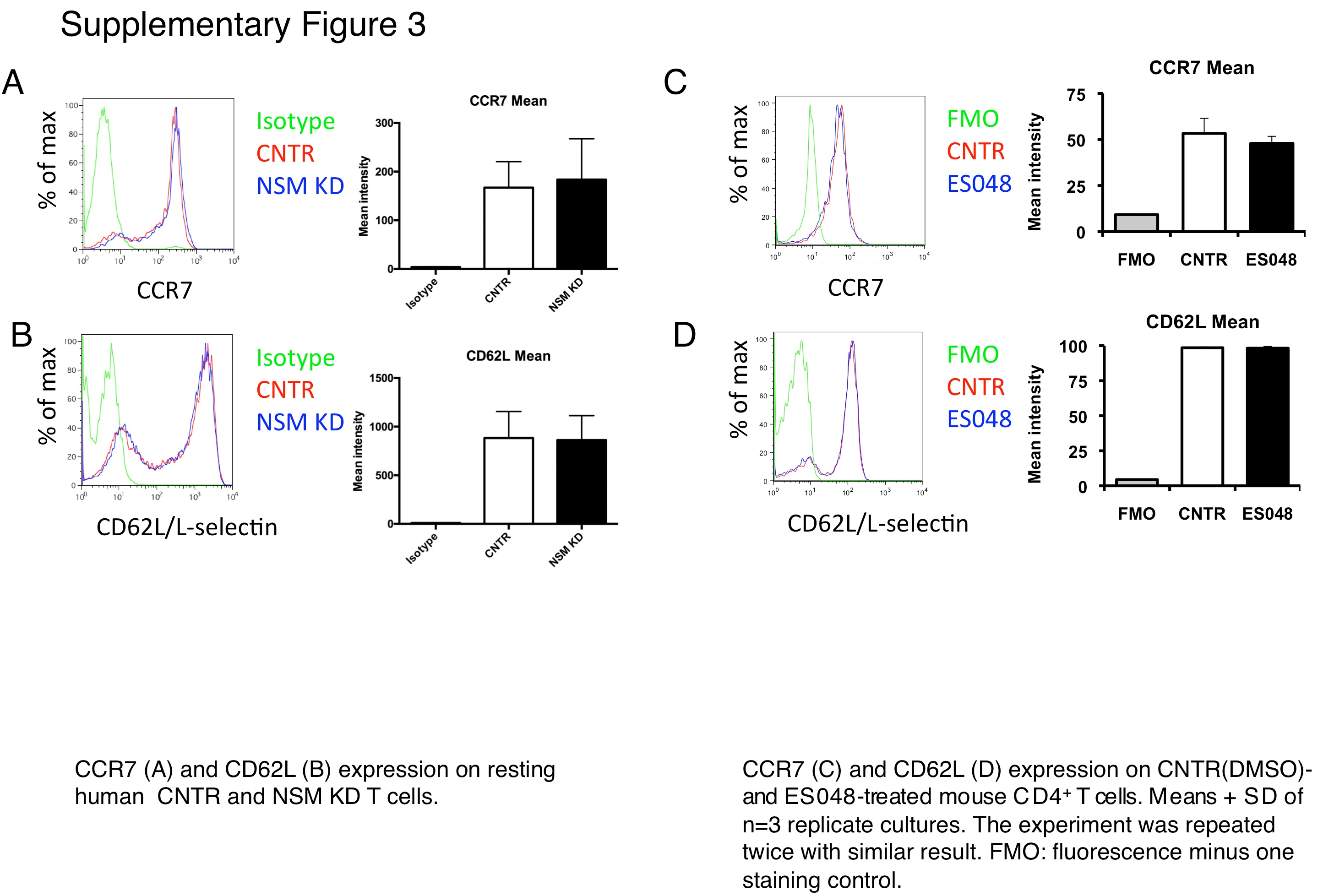

Supplement: Supplementary file 3 [file Image_3.TIF]

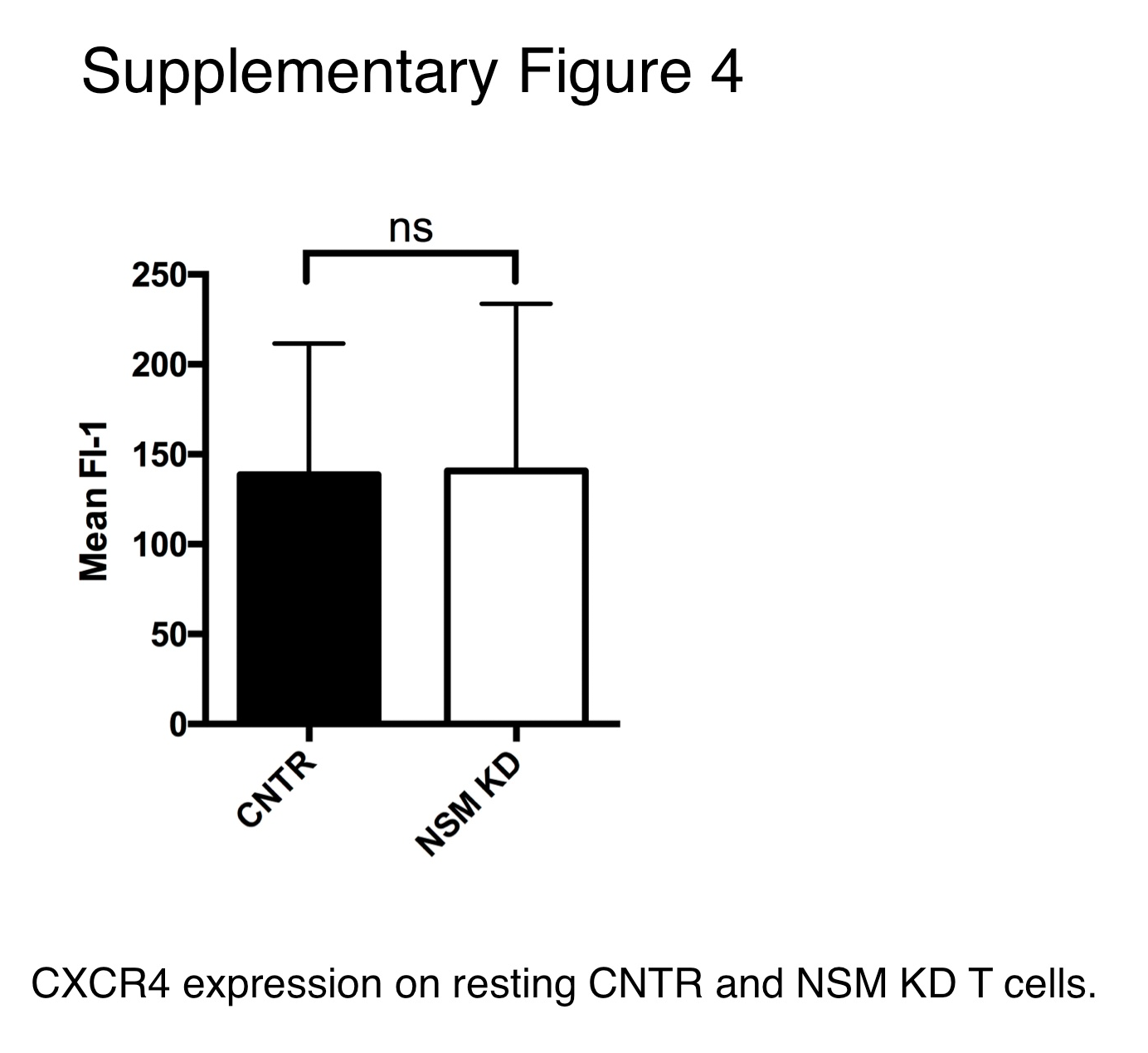

Supplement: Supplementary file 4 [file Image_4.TIF]

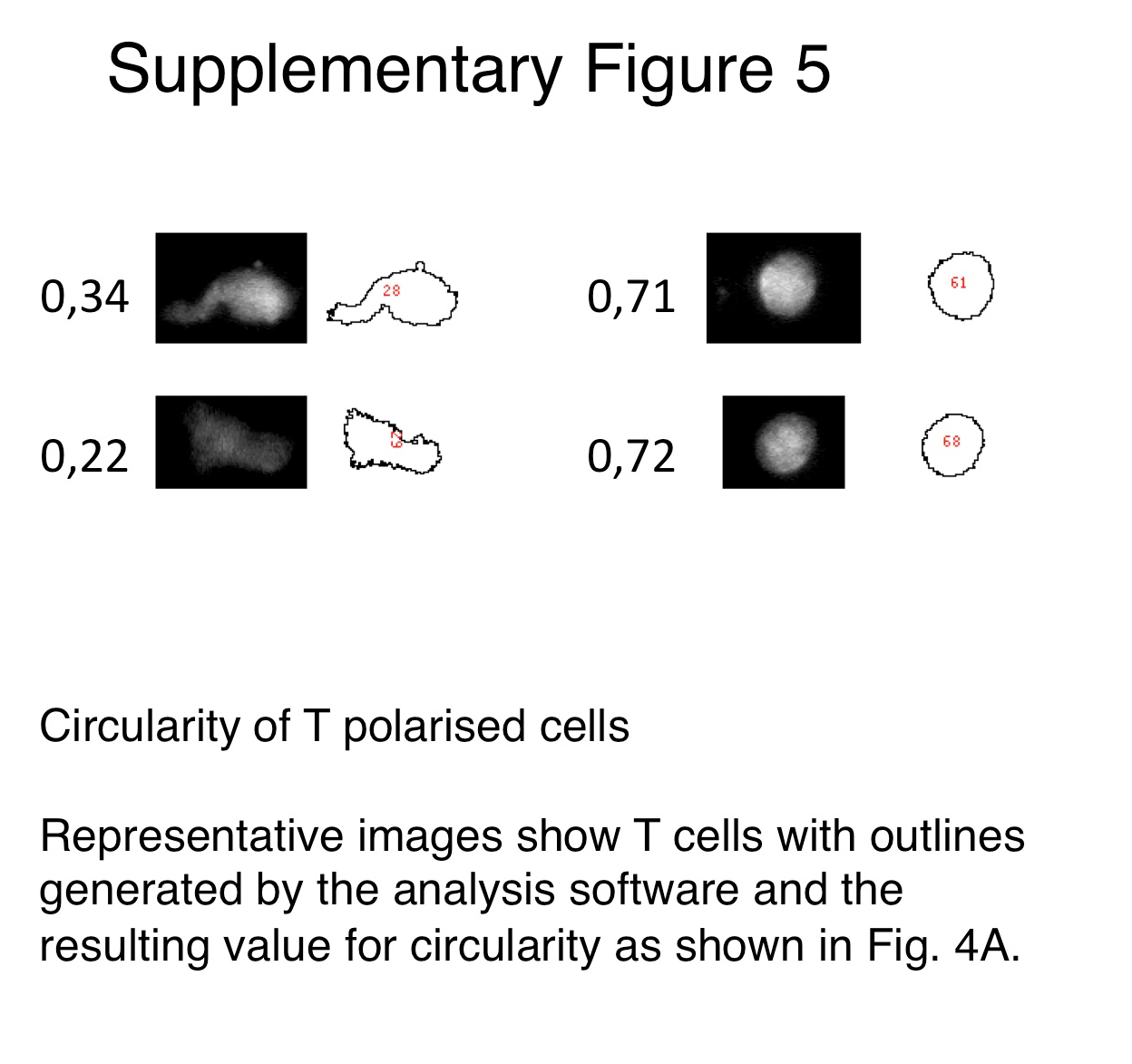

Supplement: Supplementary file 5 [file Image_5.TIF]

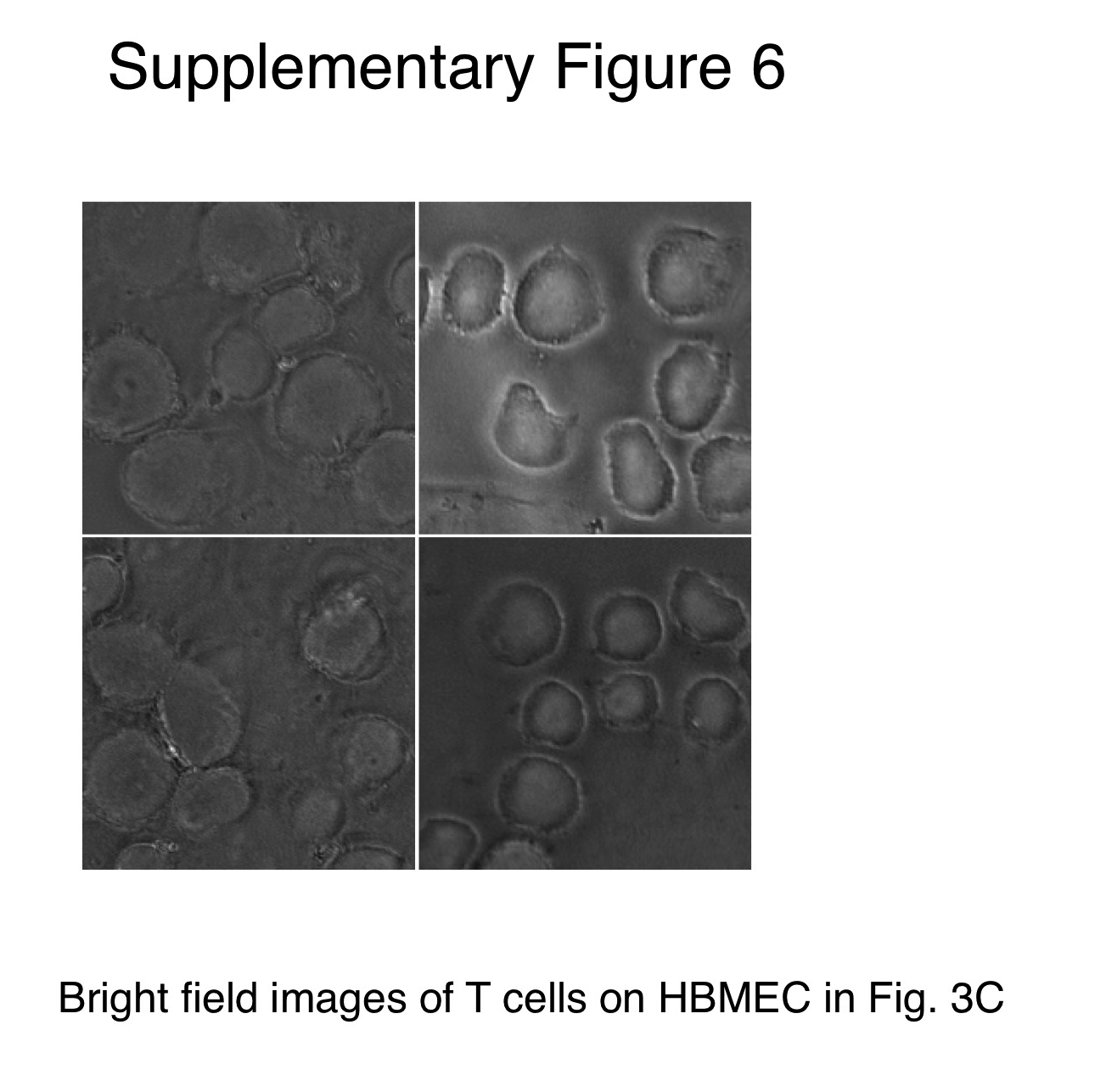

Supplement: Supplementary file 6 [file Image_6.TIF]
